# Supplementary material for: Microtubule end conversion mediated by motors and diffusing proteins with no intrinsic microtubule end-binding activity
Source: Nat Commun. 2019 Apr 11;10:1673. doi: 10.1038/s41467-019-09411-7 (PMC6459870; doi:10.1038/s41467-019-09411-7)
Supplement: Supplementary file 1 — Supplementary information [file 41467_2019_9411_MOESM1_ESM.pdf]

Microtubule end conversion mediated by motors and diffusing proteins with no intrinsic microtubule end-binding activity

Chakraborty et al.

CONTENTS

SUPPLEMENTARY NOTE 1.

DESCRIPTION OF THE MATHEMATICAL MODEL
General overview of the model
Description of model parts

Description of the MT

Description of molecular motors

Description of the diffusing MAPs

Choice of model parameters

Description of the simulation algorithm

Analysis of the simulation results

Generation of computational movies

SUPPLEMENTARY FIGURES

SUPPLEMENTARY TABLES

DESCRIPTION OF THE MATHEMATICAL MODEL

General overview of the model

A mathematical model was designed to investigate the dynamic and force-sensitive interactions between a microtubule (MT) and a multi-molecular ensemble of MT wall-binding proteins. These proteins include transporting molecular motors (kinesins) and MT-associated proteins (MAPs). The MT is modeled as a rigid rod, which is subjected to thermal motions and viscous drag. To match the geometry of our end-conversion assay, the MT-binding molecules are distributed randomly on a flat surface measuring 250 × 40 nm. The size of this patch corresponds roughly to the estimated area of the surface of a microbead, from which the molecules can reach the wall of a laterally-attached MT (Fig. 3a). A similar area of mitotic kinetochore, which has a radius of ~200 nm, engages in lateral MT binding in mammalian cells. Each molecule is firmly attached at one end to the surface of the patch, whereas its opposite end contains a MT-binding site. Molecular stalks are modeled as springs, allowing them to mediate mechanical coupling within this system. For simplicity, the maximum molecular extension length and rigidity are assumed to be the same for MAPs and motors (Supplementary Table 1). The MT-interacting ends of the molecules bind and unbind stochastically from the regularly located binding sites on the MT lattice (Fig. 3b). In addition, the motors can step unidirectionally, whereas the MAP molecules can diffuse on the MT wall (Fig. 3c). The advanced feature of our model is that all of these transitions are force-sensitive, as described in detail in the following sections. Additionally, Langevin equations are used to calculate the coordinates of all molecules and MT ends (Supplementary Figure 5a), leading to a realistic and mechanically accurate description of motions and forces arising in this molecular-mechanical system.

Description of model parts

Because comprehensive modeling of the three-dimensional interactions involving a full MT cylinder and multiple interacting molecules would be very computationally intensive, we considered a simplified one-dimensional version of the model.

Description of the MT. In the one-dimensional representation, the 13-protofilament MT is replaced by a single-protofilament MT moving along one axis. The MT is 8 μm in length, which does not change during the simulation, as in our experiments with GMPCPP-containing MTs. The position of the modeled MT is represented by the coordinate of its plus-end. The molecular binding sites form a linear array along this rod with a periodicity Δ = 4 nm. To take into account the fact that in reality, the patch-immobilized

molecules can interact with several protofilaments of the same MT cylinder, in our one-dimensional model we allow up to 5 molecules to simultaneously occupy a single binding site within the modeled linear array.

Three types of forces act on such MTs in our model: thermal forces of Brownian noise, viscous drag friction, and forces transmitted by MT-bound molecules. Thermal forces acting on the MT lead to random fluctuations in the MT end position, calculated in each iteration as:

$$\Delta x_{\mathrm{thermal}} = \sqrt{2\frac{k_{\mathrm{B}}T}{\gamma}}\ \Delta t\ N(0,1) \tag{1}$$

where Δt is the time step, γ is the viscous drag coefficient for the MT, $N(0,1)$ is a random number from a normal distribution, k_{B} is the Boltzmann constant, and T is the temperature.

Viscous drag acting on the MT is assumed to be proportional to MT velocity:

$$F_{\mathrm{viscous}} = \gamma\ \frac{x_{\mathrm{i}} - x_{\mathrm{i-1}}}{\Delta t} \tag{2}$$

where x_{i} and $x_{\mathrm{i-1}}$ are consecutive MT positions separated by time Δt.

The force acting on the MT from a MT-bound molecule is calculated as molecular stiffness $k_{\mathrm{stiffness}}$ multiplied by molecular extension.

Description of molecular motors. Kinesin is modeled with a single head that binds to MT sites stochastically with binding rate $k_{\mathrm{on}}^{\mathrm{C(k)}}$. Subsequently, the motor translocates toward the MT plus-end with stepping rate $k_{\mathrm{step}}^{\mathrm{C(k)}} = V(F)/(2\,\Delta)$, where $2\,\Delta = 8$ nm corresponds to the kinesin step size, and $V(F)$ is the velocity of motor motion under force F. The force-dependent velocity $V(F)$ for CENP-E kinesin was taken from[20] (Supplementary Table 1), for Kinesin-1 at 2 mM ATP from[54]. This experimental function was fit as in[55] using the following expression:

$$V(F) = V_{\mathrm{u}}\left(p_{\mathrm{FV}} + (1 - p_{\mathrm{FV}})e^{\frac{F\,d_{\mathrm{FV}}}{k_{\mathrm{B}}T}}\right)^{-1} \tag{3}$$

Here, V_{u} is the unloaded motor velocity, d_{FV} is the characteristic distance over which the load acts, and p_{FV} and $(1 - p_{\mathrm{FV}})$ are the fractions of biochemical and mechanical transitions in the kinesin stepping cycle, respectively.

For Kinesin-1 at for 20 μM ATP, we used the same values of p_{FV} and d_{FV} as for 2 mM ATP, as these parameters are not affected significantly by ATP concentration[54,56]. The unloaded motor velocity (parameter V_u) for Kinesin-1 at 20 μM ATP was estimated from the experimental dependency of V_{u} on ATP concentration[37].

Additionally, the model incorporates the force-dependent unbinding of motor molecules from the MT. Data for CENP-E motor unbinding rate were taken from[20]. For CENP-E, the unbinding rate $k_{\mathrm{off}}^{\mathrm{c}}$ increases exponentially with force, and this increase is symmetric for assisting and opposing loads. Unbinding rate for Kinesin-1 at 2 mM ATP concentration is based on Figure 6 in[54]. Because the unbinding rate $k_{\mathrm{off}}^{\mathrm{k}}$ for Kinesin-1 is asymmetric with respect to the direction of applied force F, for simplicity, we used a linear fit for assisting loads ($F \geq 0$), whereas an exponential fit was used for opposing loads ($F < 0$):

$$F \geq 0: \;\; k_{\mathrm{off}}^{\mathrm{k}} = k_{\mathrm{off}}^{\mathrm{ko}} + \delta^{k_{\mathrm{as}}} F$$

$$F < 0: \;\; k_{\mathrm{off}}^{\mathrm{k}} = k_{\mathrm{off}}^{\mathrm{ko}} \, e^{\frac{F\,\delta^{k_{\mathrm{op}}}}{k_{\mathrm{B}}T}} \qquad (4)$$

We also assume that $k_{\mathrm{off}}^{\mathrm{ko}}$, the unbinding rate for Kinesin-1 without any load, is same in both dependencies. Parameter $\delta^{k_{\mathrm{as}}}$ is the force sensitivity parameter for an assisting load, and $\delta^{k_{op}}$ is the characteristic distance parameter for an opposing load.

To calculate the force-unbinding function for Kinesin-1 at 20 μM ATP, we took into account the fact that the run length of Kinesin-1 is independent of ATP over a wide range of concentrations (from 2 μM to 2 mM[57]). Therefore, at low ATP concentration:

$$k_{\mathrm{off}}^{\mathrm{c(k)}}\Big|_{\mathrm{low\ ATP}} = k_{\mathrm{off}}^{\mathrm{c(k)}} \frac{V(F)|_{\mathrm{low\ ATP}}}{V(F)} \qquad (5)$$

where the "low ATP" subscript indicates the unbinding rate and force-velocity dependency at 20 μM ATP.

Finally, for all motors and conditions, the unbinding rate of the motor from the terminal binding site at the MT plus-end is assumed to be the same as its unbinding rate from the MT wall. Therefore, after the motor molecule reaches the MT plus-end, it stays bound to the end on average for the same time as it would have if it had continued walking on the MT. Under load, motor's unbinding from the MT end takes place according to the same force-dependent function as elsewhere along the MT.

Description of the diffusing MAPs. The distance between the MT-binding sites for the Ndc80 complex is Δ = 4 nm[58]. For simplicity, the same step size is used in the model for all other MAPs. To describe force-dependent transitions during a MAP's diffusion along the MT rod, we assume Bell's relationship[59]:

$$k^+ = D_o\, \Delta^{-2}\, e^{\frac{F\,\delta}{k_\mathrm{B}T}}; \qquad k^- = D_o\, \Delta^{-2}\, e^{-\frac{F\,\delta}{k_\mathrm{B}T}} \tag{6}$$

Here, k^+ and k^- are the MAP's stepping rates towards the MT plus or minus end, respectively; D_o is the diffusion coefficient along the MT for the freely diffusing MAP in the absence of external force, as measured using single-molecule TIRF-visualization *in vitro*; F is the force acting on this MAP molecule through the MT due to the MT's thermal motion, kinesin walking, and hindrance from other MT-bound MAP molecules; δ is the force-sensitivity parameter; and Δ = 4 nm is the MAP's step size.

An analogous force-dependency function was assumed for the MAP's unbinding rate from the MT wall $k_\mathrm{off}^\mathrm{M}$:

$$k_\mathrm{off}^\mathrm{M} = k_\mathrm{off}^\mathrm{Mo}\, e^{\frac{|F|\delta}{k_\mathrm{B}T}} \tag{7}$$

Here, $k_\mathrm{off}^\mathrm{Mo}$ is the MT unbinding rate for the freely diffusing MAP, and $|F|$ is the absolute value of the force acting on the MAP through the bound MT.

If a MAP reaches the last binding site at any MT end, we assume that it can detach with the same force-dependent rate as elsewhere on the MT wall ($k_\mathrm{off}^\mathrm{M}$). However, unlike the motor molecule, if the MAP does not detach immediately from the terminal binding site, it does not remain motionless at that site. Instead, the MAP continues to diffuse along the MT (Supplementary Figure 5c). From the terminal MT-binding site, the MAP can make a diffusional step towards the adjacent site with stepping rate k^- if the MAP is at the MT plus-end and k^+if the MAP is at the minus-end. However, analogous diffusional steps in the wrong direction will obviously lead to a detachment. Thus, the total unbinding rate for a MAP from the terminal binding site at any MT end is:

$$k_\mathrm{off\_end}^\mathrm{M} = k_\mathrm{off}^\mathrm{M} + \alpha k,\ \ where\ k = \begin{cases} k^+\ at\ the\ MT\ plus\ end \\ k^-\ at\ the\ MT\ minus\ end \end{cases} \tag{8}$$

Here, α is a coefficient corresponding to the probability that the MAP will take a diffusional step away from the MT end, leading to detachment.

Simulations for different MAPs were performed using the same dependencies, simulation algorithm (below), and model parameters except for two single-molecule characteristics: the rate of MT wall diffusion and residence time.

Choice of model parameters. All model parameters and corresponding values are listed in Supplementary Tables 1 and 2. An additional description is provided below.

Molecular stiffness $k_{\text{stiffness}}$ was assumed to depend on the extension length of the molecule. When molecular length exceeded 30 nm (the approximate contour length of the Ndc80 protein complex used in our *in vitro* work), high molecular stiffness of 2,000 pN μm^{-1} was assumed to prevent significant extension beyond this length under force. For shorter extensions, stiffness was assumed to be 10-fold lower, 200 pN μm^{-1}, similar to the value used in some other models featuring molecular-MT coupling[60,61]. In our one-dimensional models, this relatively low stiffness at lengths that are smaller than the resting length enables changes in the size of the linear projection of the MT-binding molecule onto the MT axis. In a real situation, the molecule can be located some distance away from the MT (Fig. 3a), so its linear projection can be much smaller than the contour length even in the absence of force. In our model, the change in the size of a linear projection takes this geometrical aspect into account, rather than corresponding to the true extension/compression under force.

Force-sensitivity parameter δ for MAPs was set to 0.2 nm, within the typical range of characteristic distances for protein–protein interactions[62].

Number of molecular motors and MAPs interacting with the MT wall. To estimate the number of MAPs bound to the MT during our end-conversion assays, we took advantage of the relationship between the number and diffusion rate of the individual MAP molecules and the resultant diffusion rate of the bound MT: when more molecules are bound, MT diffusion is slower. To define this relationship quantitatively, we carried out simulations using our model with different numbers of patch-bound MAPs (Supplementary Figure 4c). In these simulations, we used D_0 = 0.089 $\mu m^2\ s^{-1}$, corresponding to the diffusion coefficient of one Ndc80 molecule, which we determined *in vitro* for Ndc80-Broccoli on GMPCPP MTs (Supplementary Table 2). The unbinding rate for the MAPs in these simulations was assumed to be 0 to keep constant the number of the MT-bound molecules, which is therefore equal to the total number of MAPs on the patch.

The resultant MT diffusion coefficients were plotted, revealing that MT diffusion coefficient decreases hyperbolically as the number of MAPs increases (Supplementary Figure 4d).

We also derived the analytical dependency for this relationship. If all molecules within a molecular patch step independently, the stepping rate of the molecular patch is Nk^{o}, where N is the number of MAPs in the molecular patch, and $k^{\mathrm{o}} = D_{\mathrm{o}}\Delta^{-2}$ is the stepping rate of a single molecule. Displacement of the molecular patch during one step is $\Omega = \Delta/N$, where Δ is the step size for a single MAP. Therefore, the diffusion coefficient D of the molecular patch is given by:

$$D = \Omega^2 k = \frac{\Delta^2}{N^2} N k^{\mathrm{o}} = \frac{1}{N}\Delta^2 k^{\mathrm{o}} = \frac{1}{N} D_{\mathrm{o}} \tag{9}$$

This hyperbolic function is plotted on Supplementary Figure 4d (red curve) and shows a good match to numerical simulation results (black dots). These theoretical results were then compared with the MT diffusion coefficient we measured *in vitro* using coverslip-immobilized beads coated with the Ndc80 protein: $5.6 \cdot 10^{-3}$ µm^2 s^{-1}. This measured value implies that 11–13 Ndc80 complexes were bound to the MT during its diffusion (Supplementary Figure 4d, grey lines). Importantly, in simulations of MT end-retention, the number of MT-bound molecules is less than the total number of Ndc80 molecules immobilized on the molecular patch because their unbinding rate is not 0, as we assumed in simplified calculations for Supplementary Figure 4c,d. Therefore, end-retention simulations were carried out using total number of Ndc80 molecules N_{MAPs} = 15 (Supplementary Table 1). This value generates the number of MT-bound Ndc80 molecules in the 11–13 range. The same number N_{MAPs} was assumed when modeling other MAPs, as the brightness of beads coated with different MAPs was approximately similar in our *in vitro* end-conversions assays (Supplementary Figure 8). Bead coating with motors was ~3-fold brighter than with the MAPs (Supplementary Figure 8). Therefore, the number of motors in the patch in our model was $N_{\mathrm{motors}} = 45$.

Parameters of MAP diffusion on the MT wall (Supplementary Table 2). MT-wall diffusion coefficients and residence times for Ndc80, Ska1, and CLASP2 were measured using single-molecule TIRF microscopy *in vitro* under the same experimental conditions as in end-conversion assay (Supplementary Figure 7 a,b). The experimental conditions in the published studies of the diffusion of EB1 (ref[63]) and CENP-E Tail (ref[19]) were also similar.

Parameter α for the detachment of MAPs from the terminal MT-binding site. The value of this parameter for Ndc80 and other MAPs is not known. In our model, when α < 1 the probability that the MT end-bound

MAP will take a step leading to detachment is smaller than the probability of the analogous step between the adjacent MT-binding sites on the MT wall during diffusion. Thus, $\alpha < 1$ leads to an overall longer MT end association than $\alpha > 1$. To investigate this effect in more details we calculated the end-retention survival probability plots for different values of α using simulation parameters corresponding to Ndc80 and CENP-E Tail, which represent the slowest and fastest diffusing MAPs in our set (Supplementary Table 2). The faster diffusing MAP predicted poorer end-retention but the exact difference between Ndc80 and CENP-E Tail depended on α. We calculated the difference between the predicted survival probability for MT ends at 30 min from the start of simulations for these two MAPs. With increasing α, this difference increased and came to a plateau at $\alpha = 10^{-2}$, implying that model predictions for end-retention are no longer sensitive to this parameter (Supplementary Figure 5d). Accordingly, this value was used for simulations with all MAPs. Relatively good agreement between the resultant predictions and experimental measurements for these MAPs suggest that the value of α is indeed similar for these proteins. The mismatch for Ska1 complex could be explained by a different value of α for this protein or more trivial factors, e.g., partial inaccessibility of the Ska1 microtubule-binding site after Ska1 is conjugated to the bead surface. This would lead to a shorter end-retention time in experiment than in the model, which does not include such complexities. Future work using single-molecule visualization at MT ends is needed to determine the experimental values of α for different MAPs.

Description of the simulation algorithm

At the start of each calculation, the positions of all MAPs and kinesin motors along the patch-representing linear segment were chosen randomly. The initial MT configuration was centered relative to this segment, and calculations started with all molecules not bound to the MT. Subsequent iterations were carried out with the time step Δt for total simulation time t_{total}. For each stochastic event E occurring with rate k, the probability Ψ_{E} of this event to occur at each time step was calculated as in[64]:

$$\Psi_{\mathrm{E}} = 1 - e^{-k\Delta t} \qquad (10)$$

Next, a random number p from the range [0, 1] with constant probability density was generated. If p was smaller than Ψ_{E}, the corresponding stochastic event E was assumed to be accomplished.

Four types of stochastic events were calculated at each step:

(1) Binding of MAPs and motors to sites on the MT. For each unbound molecule, the linear distance between its surface-attached end and the nearest binding site on the MT was calculated. If the distance

to the nearest MT site was ≤ 2 nm, the MAP (or motor) molecule was assumed to interact with this site with the binding rate k^{M}_{on} (or k^{k}_{on}).

(2) Unbinding of molecular motors and MAPs from the MT. For each MT-bound molecule, the applied force *F* was calculated as molecular stiffness $k_{\mathrm{stiffness}}$ multiplied by the molecular extension length, calculated as the linear distance between the surface-bound and MT-bound ends of the molecule (corresponding to the length projection in three-dimensional space). Each MT-bound molecule detached according to force-dependent unbinding rates, as described in the sections for MAPs and kinesins.

(3) MT-dependent translocation of molecules. The stepping of kinesin motors and diffusion of MAPs was calculated according to the force-dependent transition rates described in the corresponding sections.

(4) Motion of the MT. The coordinate of the new MT plus-end position $x_{\mathrm{i}}^{\mathrm{MT}}$ was calculated using the Langevin equation:

$$x_{\mathrm{i}}^{\mathrm{MT}} = x_{\mathrm{i-1}}^{\mathrm{MT}} + \frac{\Delta t}{\gamma} F_{\mathrm{total}} + \sqrt{2\frac{k_{\mathrm{B}}T}{\gamma}}\ \Delta t\ N(0,1) \qquad (11)$$

where $x_{\mathrm{i-1}}^{\mathrm{MT}}$ is the MT plus-end position at the previous time step, Δt is the time step, γ is the viscous drag friction coefficient for the MT, $N(0,1)$ is a random number from a normal distribution, k_{B} is the Boltzmann constant, T is the temperature, and F_{total} is the total force applied to the MT from all MT-bound MAPs and motors.

F_{total} was calculated using the following expression:

$$F_{\mathrm{total}} = \sum_{m=1}^{N_{\mathrm{boundMAPs}}} \Delta l_{\mathrm{m}} k_{\mathrm{stiffness}} + \sum_{n=1}^{N_{\mathrm{boundKinesins}}} \Delta l_{\mathrm{n}} k_{\mathrm{stiffness}}^{\mathrm{Kinesin}} \qquad (12)$$

where Δl_{m} corresponds to the extensions of each MAP bound to the MT, and Δl_{n} corresponds to the extensions of each kinesin bound to MT; and $N_{\mathrm{boundMAPs}}$ and $N_{\mathrm{boundKinesins}}$ are the total numbers of MT-bound MAPs and motors at this time step, respectively.

After these stochastic events were calculated and MT position was updated, the positions of all binding sites on the MT relative to the surface-bound molecules were also updated, yielding the updated molecular extension lengths and forces acting on each molecule. The simulation time was increased by Δt and compared with t_{total}: if the simulation time was less than t_{total}, the algorithm was repeated from step (1); if the simulation time was greater than t_{total}, the simulation was stopped.

Analysis of the simulation results

To obtain the end-retention survival probability plot from model simulations, we carried out simulations for t_{total} = 30 min, corresponding to the duration of experimental observations. Survival time was defined as the first-time step from the start of simulations for which the MT had zero bound MAPs and motors. The end-retention survival probability curve was then plotted from the fraction of MTs that had at least one bound molecule (either a motor or a MAP) at each time step, based on 32 independent simulations for each pair of motors and MAPs.

Generation of computational movies

To generate three-dimensional computational movies, calculations were carried out using a one-dimensional model, as described in the simulation algorithm. Then, image frames were generated using a custom-designed program written in Mathematica to represent three-dimensional interactions. Briefly, a single protofilament was complemented with tubulin-representing spheres to form a full cylinder 0.9 µm long, and then positioned 0.02 µm above the patch surface. Positions of the surface-bound ends of each molecule along the MT axis were as in the original one-dimensional simulation, whereas their positions in the MT-perpendicular direction were chosen randomly within a 45-nm distance from the protofilament, mimicking a two-dimensional patch with attached molecules. Translocation of each molecule along the MT was assumed to take place along the nearest MT protofilament, and the coordinates of the MT-bound ends of all molecules at each time point were taken from the corresponding one-dimensional simulation. To improve visual discrimination of the MT-bound and -unbound molecules, the latter are shown in the movies in random orientations. Furthermore, to avoid cluttering, the number of molecules in these simulations was reduced relative to our standard simulations: N_{MAPs} = 5, N_{motor} = 5. Other parameters used in movie simulations: k_{on} = 3.6 s^{-1}, k_{on} = 120 s^{-1}, MAP diffusion coefficient D_{o} = 0.0038 $\mu m^2\ s^{-1}$.

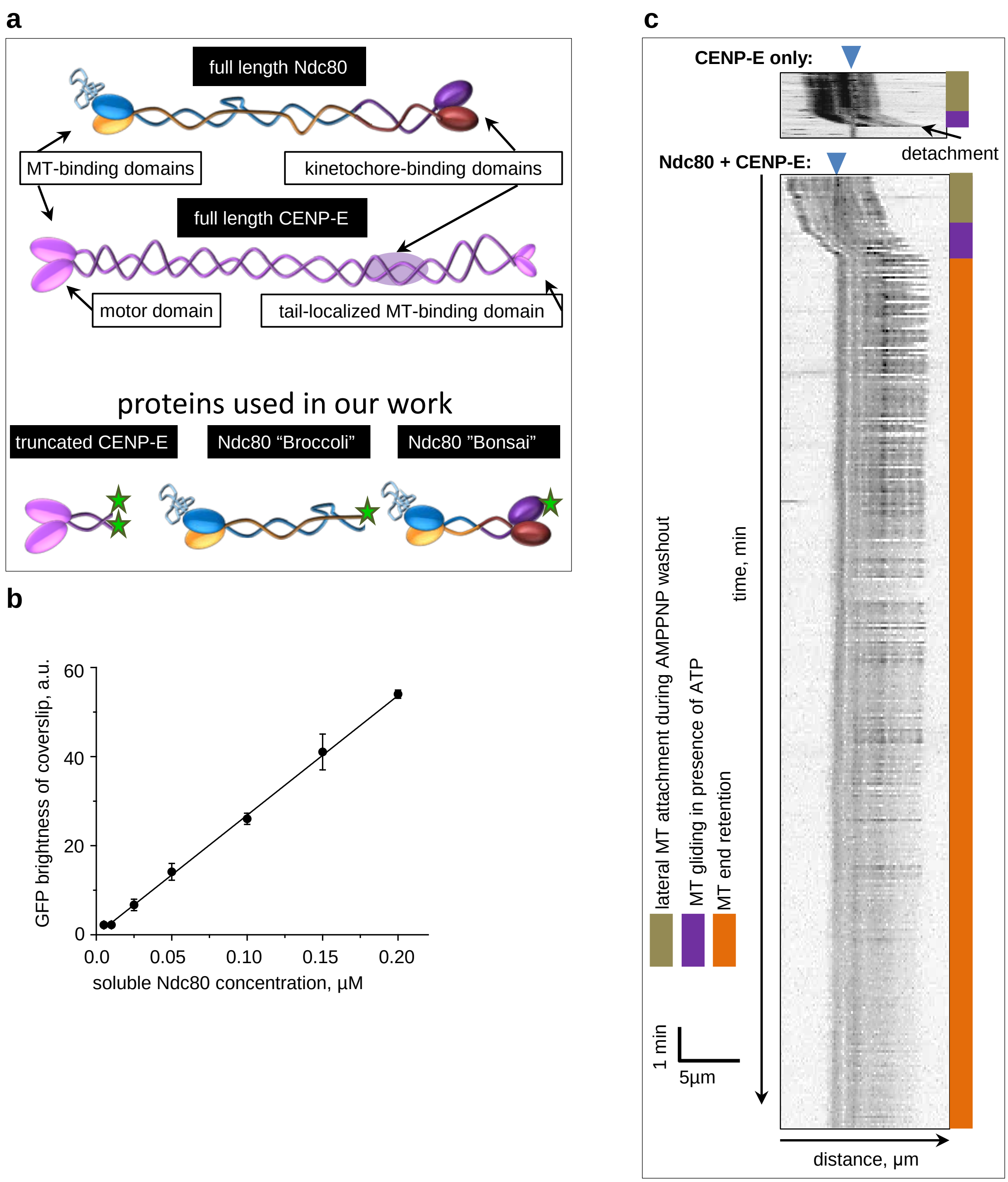

Supplementary Figure 1. MT gliding on coverslips and beads.

(a) Schematic of the full-length Ndc80 and CENP-E proteins, and the truncated protein constructs used in this study.

(b) Quantification of coverslip brightness in MT gliding experiments that used a mixture of proteins: Myc-tagged CENP-E with no fluorescent label and GFP-labeled Ndc80 Bonsai. The latter protein was added to the microscopy chamber at the indicated concentrations; the unbound proteins were removed; and the GFP fluorescence brightness of the coverslip coating was measured. Data points and error bars indicate means ± SEM of *N* = 3 independent experiments. Source data are provided as a Source Data file.

(c) Representative kymographs of motions of bead-bound stabilized MTs in the presence of ATP. Arrowheads indicate positions of coverslip-immobilized beads. Colored bars provide interpretations for the kymographs (see labels).

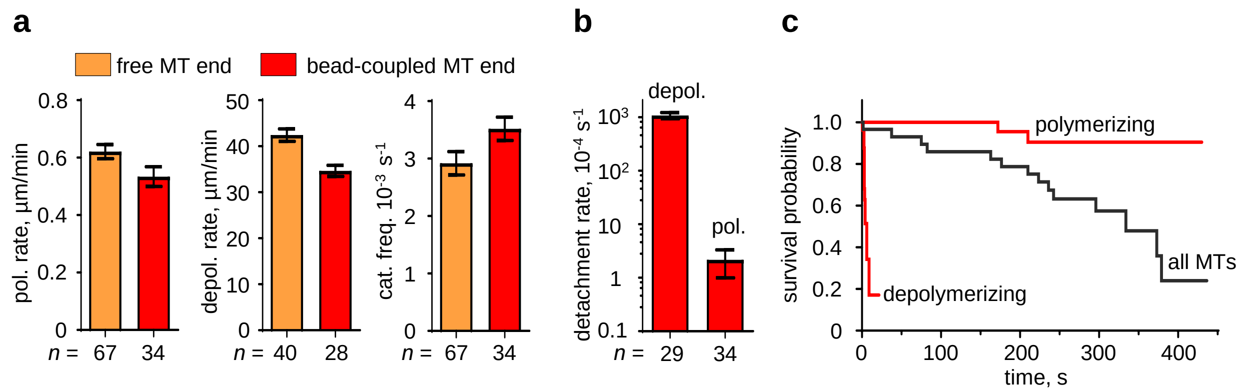

Supplementary Figure 2. Behavior of MT ends in assays with freely floating beads. MT end coupling in experiments using MTs grown from coverslip-immobilized seeds and the freely floating Ndc80+CENP-E beads (prepared in a 1:4 ratio). Beads randomly attached to MT walls, become transported to the MT plus-end by CENP-E motor and travelled at the ends of dynamic MTs. Source data are provided as a Source Data file.

(a) Dynamics of MT ends that are coupled to the beads or not coupled (i.e., free MT ends). Columns and error bars indicate means ± SEM of total number of examined MT ends (indicated under each column) from 2 independent experiments. To estimate SEM for catastrophe frequency, bootstrapping was performed using Excel: data from all MTs were pulled together, and 10 random points were drawn with replacement 10 times. These graphs show that presence of the beads does not affect significantly the rates of MT polymerization and depolymerization, as well as the catastrophe frequency.

(b) Frequency of bead detachment from the ends of dynamic MTs was calculated as inverse of the ratio of total time beads were bound at the MT ends to the number of detachment events (total number of examined MT ends is indicated under each column). SEM was estimated using same procedure as for catastrophe frequency in panel a. This graph shows that bead coupling is more stable at polymerizing MT ends than on depolymerizing ends.

(c) Survival plot of attachment time for beads coupled to the MT ends. Graphs are based on the same data set as in panel (b).

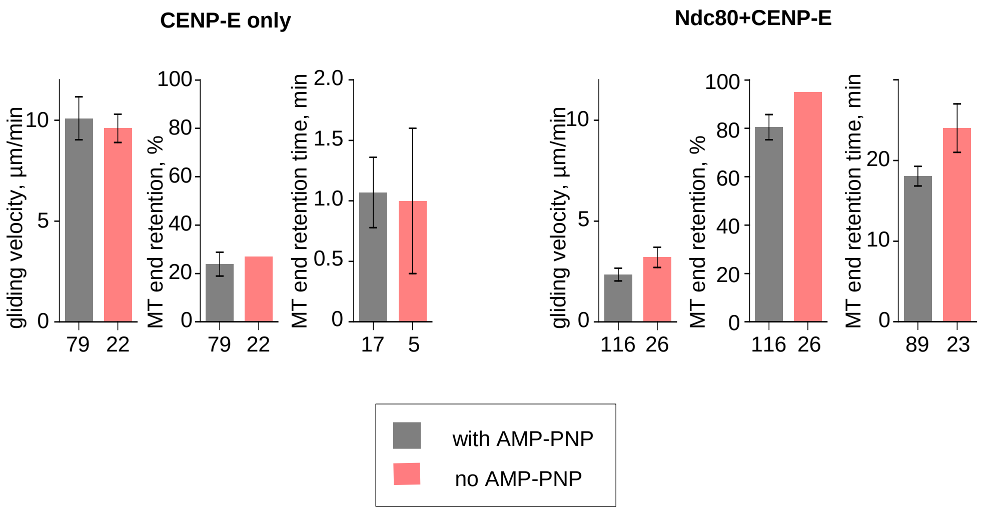

Supplementary Figure 3. Preincubation with AMP-PNP does not affect the outcome of end-conversion assay.

Results from experiments in which stabilized MTs attached to the coverslip-immobilized beads via random collisions in the buffer with ATP or in the presence of AMP-PNP, which was subsequently replaced with ATP. Because no significant differences were observed between these different assay conditions, these data sets were combined. Grey columns show means ± SEM, N = 3 and 6 independent experiments for CENP-E only and Ndc80+CENP-E samples; total number of examined MTs is shown under each column. Pink columns show mean ± SEM for the total number of examined MTs indicated under each column. Source data are provided as a Source Data file.

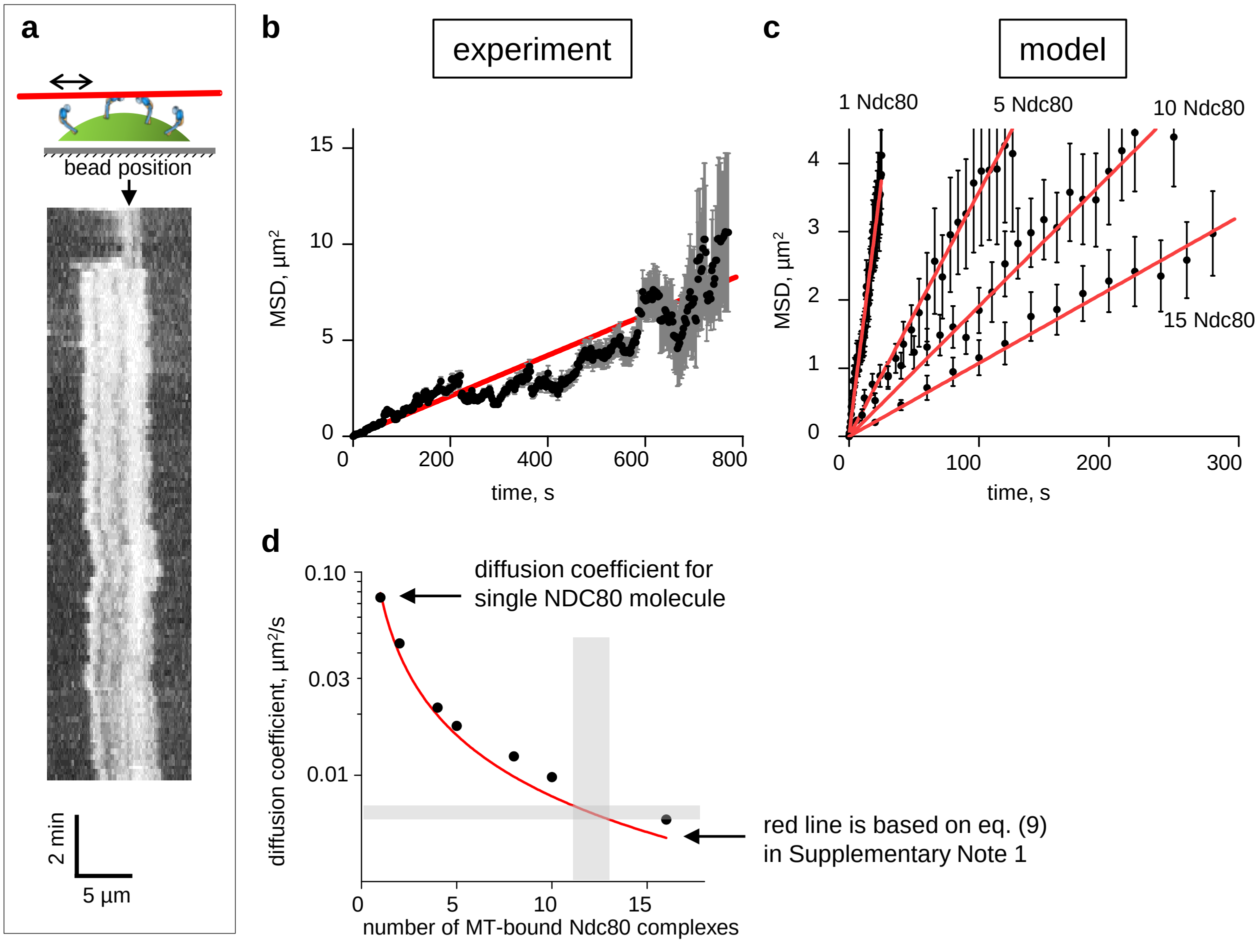

Supplementary Figure 4. Analysis of the diffusion of MTs mediated by multiple Ndc80 molecules.

(a) Schematic of an experiment in which stabilized MTs diffused on the surface of Ndc80-coated beads. Representative kymograph shows one diffusing MT. The position of the coverslip-immobilized bead is marked with a black arrow.

(b) MSD of diffusing MTs vs. time is based on tracings of 20 MTs observed in 2 independent experiments. Red line is the linear fit. Error bars are SEM.

(c) Theoretically predicted MSD of MTs diffusing on molecular patches with different numbers of MT-bound Ndc80 molecules. Error bars are SEM. Source data for panels (b)-(d) are provided as a Source Data file.

(d) Theoretical relationships between diffusion coefficient and the number of MT-bound Ndc80 molecules. Black dots are data from numerical simulations, and the red line is the prediction based on analytical results. Shaded horizontal bar corresponds to the experimentally measured MT diffusion coefficient. Shaded vertical bar corresponds to the estimated range of the number of MT-bound Ndc80 molecules.

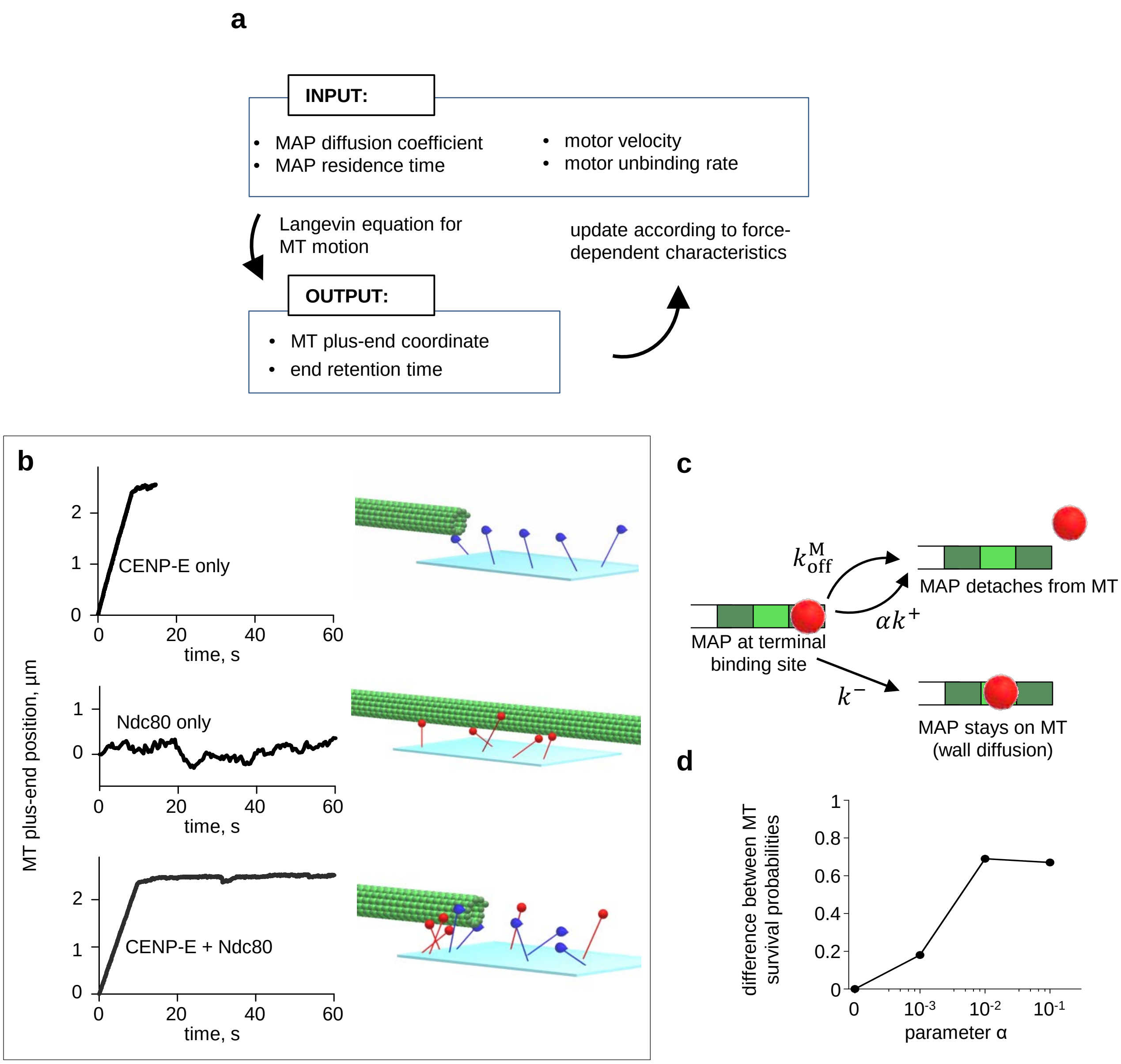

Supplementary Figure 5. Mathematical modeling of MT end conversion.

(a) Schematic of computational flow in the model. Input parameters and dependencies were used to calculate the coordinates of MT plus-ends. Forces acting on each MAP and motor molecules were then calculated to determine the probabilities of molecular stepping (diffusional or directional) and unbinding from the MT.

(b) Representative trajectories for MT plus-ends (left) calculated for molecular patches with different molecular compositions (right).

(c) Schematic illustration of the behavior of a MAP molecule at the terminal binding site of the MT. The molecule can step away from the MT end, as during wall diffusion, or can detach from the end via two independent pathways (see text for details).

(d) MT end-retention outcome as a function of the parameter α. Vertical axis shows difference between survival probabilities predicted by the model for two protein pairs: CENP-E motor with CENP-E tail and CENP-E motor with Ndc80 (see "Choice of model parameters" in the Supplementary Note 1 for details).

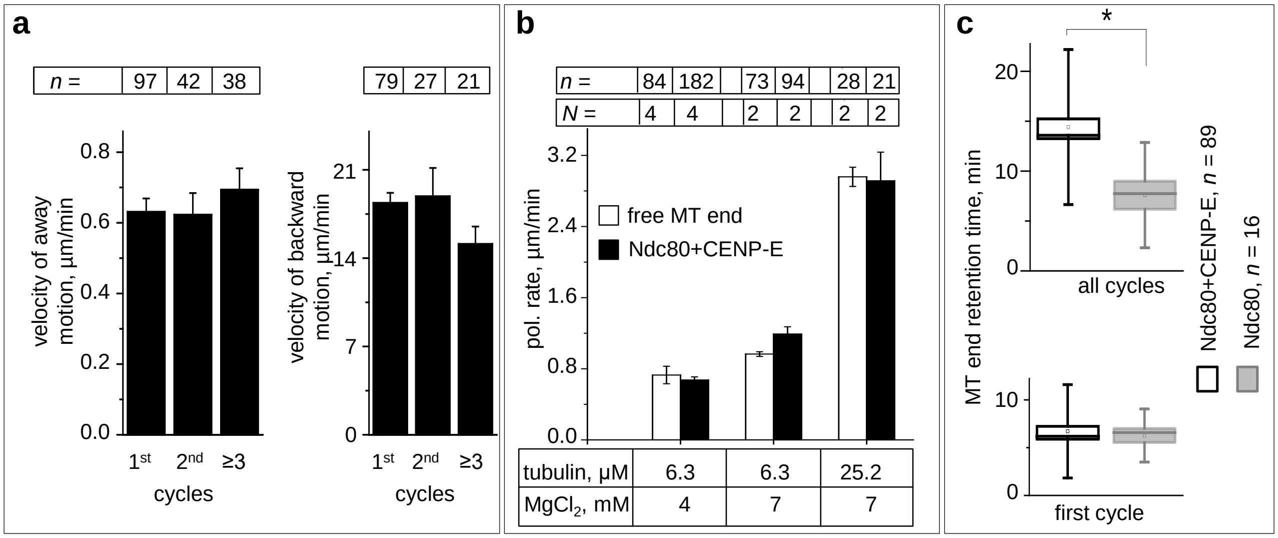

Supplementary Figure 6. Dynamics of coupled MT ends.

(a) Results from an end-conversion assay using Ndc80+CENP-E beads and soluble tubulin. The graph shows the velocity with which labeled MT fragment moved away and toward the bead. Columns are medians ± SEM for data from *N* = 4 independent experiments with *n* observed MTs, as indicated above each column. Velocities were similar during repeated MT dynamics cycles, consistent with the idea that tubulin dynamics are the driving force. Source data for panels (a)-(c) are provided as a Source Data file.

(b) Rate of polymerization of bead-free MT plus-ends and the velocity with which labeled MT fragments moved away from beads coated with Ndc80 and CENP-E. Columns are means ± SEM for *N* = 4 independent experiments and means ± SD (determined by bootstrap statistical analysis) for *N* = 2 experiments. Polymerization of free MT ends and the bead-coupled ends was affected similarly by different concentrations of soluble tubulin and $MgCl_2$.

(c) Duration of dynamic attachment for the first cycle or all cycles of MT end coupling by beads coated with Ndc80, with or without CENP-E. Box is median ± SEM and whiskers are SDs; data are combined from *N* = 4 (Ndc80+CENP-E) or *N* = 6 (Ndc80) independent trials. Significance of differences between two data sets was evaluated using the Mann–Whitney test; * = $p < 0.05$. The duration of the first cycle is same for these bead coatings, but the total duration was significantly reduced in the absence of CENP-E. Source data are provided as a Source Data file for all panels.

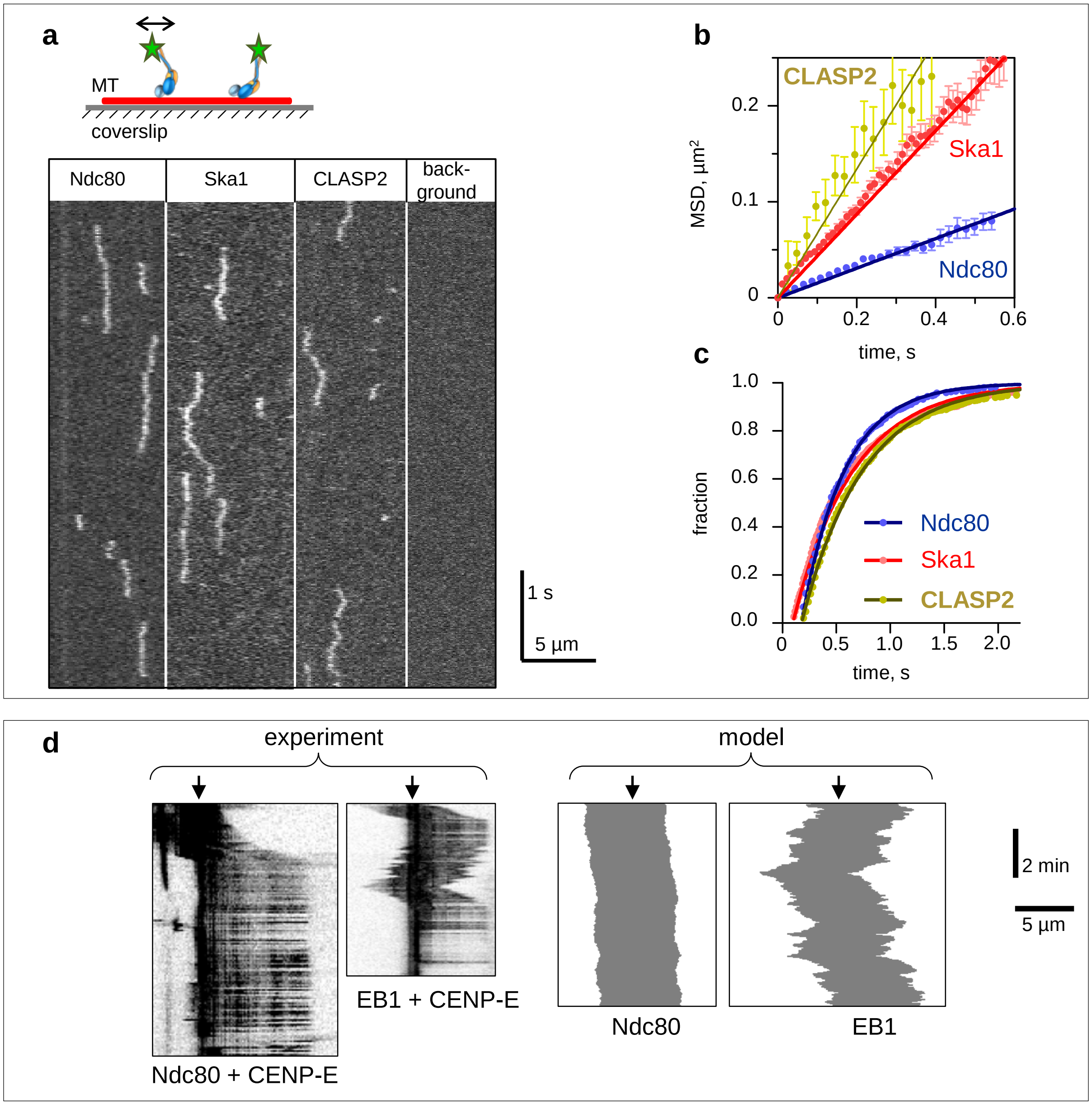

Supplementary Figure 7. Diffusion of single MAP molecules measured in vitro and the diffusional behavior of MTs in end conversion assay.

(a) Schematic of an experiment and representative kymographs depicting diffusion of single molecules of indicated MAPs, visualized via TIRF microscopy. Background image was obtained in an area with no MTs.

(b) MSD of single-molecule diffusion. Error bars are SEM, see Supplementary Table 2.

(c) Cumulative distributions of residence times during diffusional events for the indicated MAPs. Source data for panels (b) and (c) are provided as a Source Data file.

(d) Experimental and theoretical analyses of the diffusional behavior of MTs in end conversion assay following the loss of MT tip attachment (i.e., end-to-wall transition). Experimental kymographs show MTs gliding on the coverslip-immobilized beads coated with CENP-E and either Ndc80 or EB1. In EB1 presence, after the MT achieved wall-to-end transition, it suddenly exhibited wall diffusion on the bead. After the new round of directed transport and establishment of new end-attachment, the MT end again loses this configuration and starts diffusing. Theoretical kymographs on the right show predicted behavior of the end-bound MTs after the CENP-E-dependent transport was turned off: the MT begins to diffuse vigorously on the EB1-containing patch but not on the patch with Ndc80. Arrows indicate bead/patch positions.

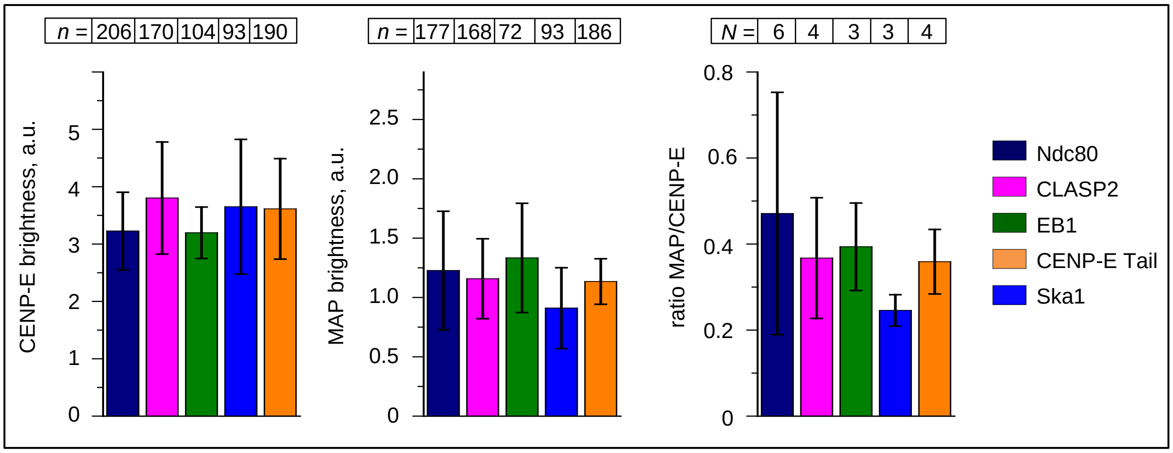

Supplementary Figure 8. Density of bead coating by different proteins.

GFP brightness of beads coated with different proteins is shown on first two graphs (see Methods for details). Last graph shows the ratio of GFP brightness of the indicated MAP to that of CENP-E kinesin on the same beads. Bars represent means ± SEM for $N \geq 3$ independent experiments in which a total of *n* beads were examined. Source data are provided as a Source Data file .

SUPPLEMENTARY TABLE 1

PARAMETERS USED IN THE SIMULATIONS

**PART A. General model parameters**			
**Symbols**	**Description**	**Value**	**Comments**
Δt	simulation time step	4 µs	chosen for convergence of simulation algorithm
t_{total}	total simulation time	30 min	as in experiments *in vitro*
L_{MT}	length of microtubule	8 µm	typical MT length in experiments *in vitro*
γ	viscous drag coefficient per microtubule length	0.014 pN·s·µm^{-2}	calculated based on dynamic viscosity (0.002 Pa s) and diameter of MT (25 nm)
$k_{\mathrm{stiffness}}$	stiffness of MAPs and motors	extension ≤ 30 nm: 200 pN µm^{-1}<br>extension > 30 nm: 2,000 pN µm^{-1}	see “Choice of model parameters” in the Supplementary Note 1
$k_{\mathrm{B}}T$	energy scale factor	4.11 pN·nm	

SUPPLEMENTARY TABLE 1 (continued)

PART B. Parameters and functions describing MAPs			
**Symbols**	**Description**	**Value**	**Comments**
D_{o}	diffusion coefficient for single MAP molecule on the MT	varied to model different MAPs	measured *in vitro* (see Supplementary Table 2)
τ	MAP residence time on the MT during diffusion	varied to model different MAPs	measured *in vitro* (see Supplementary Table 2)
Δ	MAP step size during diffusion	4 nm	assumed to be the same for all MAPs
δ	force-sensitivity parameter	0.2 nm	see "Choice of model parameters" in the Supplementary Note 1
$k_{\mathrm{on}}^{\mathrm{M}}$	rate of MAP binding to the MT	20 s^{-1}	see section "Choice of model parameters" in the Supplementary Note 1
α	probability that the MAP will take a diffusional step away from the MT at terminal binding site	10^{-2}	see section "Choice of model parameters" in the Supplementary Note 1
N_{MAPs}	number of MAPs in the molecular patch	15	see section "Choice of model parameters" in the Supplementary Note 1
$k_{\mathrm{off}}^{\mathrm{Mo}}$	MAP unbinding rate from the MT wall without load	1 / τ	
k^{+}	MAP stepping rate towards the MT plus end		see eq. (6) in the Supplementary Note 1
k^{-}	MAP stepping rate towards the MT minus end		see eq. (6) in the Supplementary Note 1
$k_{\mathrm{off}}^{\mathrm{M}}$	MAP unbinding rate from the MT wall under external force		see eq. (7) in the Supplementary Note 1
$k_{\mathrm{off\_end}}^{\mathrm{M}}$	MAP unbinding rate from the MT terminal binding site		see eq. (8) in the Supplementary Note 1

SUPPLEMENTARY TABLE 1 (continued)

PART C. Parameters and functions describing motors					
**Symbols**	**Description**	**Value**			**Comments**
		CENP-E	Kinesin-1 2 mM ATP	Kinesin-1 20 μM ATP	
V_{u}	velocity of unloaded motor	0.26 μm s^{-1}	0.62 μm s^{-1}	0.1 μm s^{-1}	see "Description of molecular motors" in the Supplementary Note 1
d_{FV}	force-sensitivity parameter	2.8 nm	5.3 nm	5.3 nm	
p_{FV}	fraction of biochemical transitions in kinesin stepping cycle	0.58	0.99	0.99	
$k_{\mathrm{on}}^{\mathrm{C(k)}}$	rate of motor binding to the MT	0.4 s^{-1}			see "Choice of model parameters" in the Supplementary Note 1
$k_{\mathrm{step}}^{\mathrm{C(k)}}$	motor stepping rate toward the MT plus-end	$V(F)/(2\,\Delta)$			
$k_{\mathrm{off}}^{\mathrm{Co}}$	unbinding rate for CENP-E without load	0.12 s^{-1}			data from[20]
δ^{C}	force-sensitivity parameter for CENP-E unbinding	2 nm			
$k_{\mathrm{off}}^{\mathrm{ko}}$	unbinding rate for Kinesin-1 without load	1.1 s^{-1} for 2 mM ATP<br>0.11 s^{-1} for 20 μM ATP			see "Description of molecular motors" in the Supplementary Note 1
$\delta^{\mathrm{k_{op}}}$	force-sensitivity parameter for Kinesin-1 unbinding under opposing load	0.6 nm			
$\delta^{\mathrm{k_{as}}}$	force-sensitivity parameter for Kinesin-1 unbinding under assisting load	1.5 $pN^{-1}\cdot s^{-1}$ for 2 mM ATP<br>0.15 $pN^{-1}\cdot s^{-1}$ for 20 μM ATP			
N_{motors}	number of motors in the molecular patch	45			see "Choice of model parameters" in the Supplementary Note 1

SUPPLEMENTARY TABLE 2

MOLECULAR PARAMETERS OF MAP-MT INTERACTIONS MEASURED USING SINGLE-MOLECULE TIRF MICROSCOPY.

N – number of independent experiments; *n* – number of single molecules analyzed. For experiments with $N \geq 3$, errors are SEM; for $N = 2$, errors are 95% confidence intervals.

MAP	Ndc80	Ska1	CLASP2	CENP-E Tail†	EB1¶
Diffusion coefficient (*D*), $\mu m^2\ s^{-1}$	0.089 ± 0.003 ($N = 2$, $n = 1037$)	0.23 ± 0.01 ($N = 3$, $n = 2018$)	0.33 ± 0.02 ($N = 4$, $n = 558$)	1.6	0.31 ± 0.01
Residence time (τ), s	0.43 ± 0.01 ($N = 2$, $n = 551$)	0.61 ± 0.02 ($N = 2$, $n = 974$)	0.65 ± 0.01 ($N = 2$, $n = 518$)	0.47 ± 0.03	0.08 ± 0.01

† data from[19]

¶ data from[63]
