# Supplementary material for: Microtubule end conversion mediated by motors and diffusing proteins with no intrinsic microtubule end-binding activity
Source: Nat Commun. 2019 Apr 11;10:1673. doi: 10.1038/s41467-019-09411-7 (PMC6459870; doi:10.1038/s41467-019-09411-7)
Supplement: Supplementary file 3 — Reporting Summary [file 41467_2019_9411_MOESM3_ESM.pdf]

nature research

Corresponding author(s): *Double-blind peer review submissions: write DBPR and your manuscript number here instead of author names.*

Reporting Summary

Nature Research wishes to improve the reproducibility of the work that we publish. This form provides structure for consistency and transparency in reporting. For further information on Nature Research policies, see Authors & Referees and the Editorial Policy Checklist.

Statistical parameters

When statistical analyses are reported, confirm that the following items are present in the relevant location (e.g. figure legend, table legend, main text, or Methods section).

n/a	Confirmed	
☐	☒	The exact sample size (*n*) for each experimental group/condition, given as a discrete number and unit of measurement
☐	☒	An indication of whether measurements were taken from distinct samples or whether the same sample was measured repeatedly
☐	☒	The statistical test(s) used AND whether they are one- or two-sided<br>*Only common tests should be described solely by name; describe more complex techniques in the Methods section.*
☒	☐	A description of all covariates tested
☒	☐	A description of any assumptions or corrections, such as tests of normality and adjustment for multiple comparisons
☐	☒	A full description of the statistics including central tendency (e.g. means) or other basic estimates (e.g. regression coefficient) AND variation (e.g. standard deviation) or associated estimates of uncertainty (e.g. confidence intervals)
☒	☐	For null hypothesis testing, the test statistic (e.g. *F*, *t*, *r*) with confidence intervals, effect sizes, degrees of freedom and *P* value noted<br>*Give P values as exact values whenever suitable.*
☒	☐	For Bayesian analysis, information on the choice of priors and Markov chain Monte Carlo settings
☒	☐	For hierarchical and complex designs, identification of the appropriate level for tests and full reporting of outcomes
☒	☐	Estimates of effect sizes (e.g. Cohen's *d*, Pearson's *r*), indicating how they were calculated
☐	☒	Clearly defined error bars<br>*State explicitly what error bars represent (e.g. SD, SE, CI)*

Our web collection on statistics for biologists may be useful.

Software and code

Policy information about availability of computer code

Data collection	Microscopy images were captured using NIS elements software (Nikon) and Metamorph (Molecular Devices)
Data analysis	Image analysis was done using ImageJ (NIH Image) and Metamorph (Molecular Devices). Graphs were prepared using Origin9 (OriginLab) and Prism7 (GraphPad) software. Statistical tests were carried out using Origin9. Model algorithm was implemented using Mathematica (Wolfram Research)

For manuscripts utilizing custom algorithms or software that are central to the research but not yet described in published literature, software must be made available to editors/reviewers upon request. We strongly encourage code deposition in a community repository (e.g. GitHub). See the Nature Research guidelines for submitting code & software for further information.

Data

Policy information about availability of data

All manuscripts must include a data availability statement. This statement should provide the following information, where applicable:
- Accession codes, unique identifiers, or web links for publicly available datasets
- A list of figures that have associated raw data
- A description of any restrictions on data availability

Authors declare that all data in support of the findings of this paper are provided within the main and supplementary part of the manuscript. Data for individual

trials, images and other raw data will be provided upon request. All model assumptions, equations, parameter values and simulation algorithm are provided in the Supplement.

Field-specific reporting

Please select the best fit for your research. If you are not sure, read the appropriate sections before making your selection.

☒ Life sciences ☐ Behavioural & social sciences ☐ Ecological, evolutionary & environmental sciences

For a reference copy of the document with all sections, see nature.com/authors/policies/ReportingSummary-flat.pdf

Life sciences study design

All studies must disclose on these points even when the disclosure is negative.

Sample size	Individual trials for in vitro assays were analyzed fully, so their sample sizes were limited only by the number of observed events. The trials were repeated several times to ensure reproducibility. The resultant sample sizes (number of independent trials and total number of observed events) are noted in figure legends
Data exclusions	All events were scored unless they could not be analyzed fully due to technical limitations, as described in corresponding Methods sections
Replication	All experimental observations were repeated several times, collecting data from multiple beads or microtubule ends for each condition. Replications (also called independent trials) involved preparation of new microscopy chambers with fresh buffers and protein aliquotes, as well as conducting observations on different days. Key experiments were repeated by two independent researchers (end conversion with Ndc80, CENP-E and Kinesin-1). Because variability within data set from an independent trial is usually lower than between the trials, data were presented as averages of means from different trials and difference between such replications were represented with SEMs. In some cases, data from all replications were pooled, and average for the combined data set was reported with errors described in figure legends (SD, SEM or estimated with bootstrapping).
Randomization	No randomization were done
Blinding	Blinding was not performed because it was difficult to carry out experimental and control assays in parallel

Reporting for specific materials, systems and methods

Materials & experimental systems

n/a	Involved in the study	
☒	☐	Unique biological materials
☐	☒	Antibodies
☒	☐	Eukaryotic cell lines
☒	☐	Palaeontology
☒	☐	Animals and other organisms
☒	☐	Human research participants

Methods

n/a	Involved in the study	
☒	☐	ChIP-seq
☒	☐	Flow cytometry
☒	☐	MRI-based neuroimaging

Antibodies

Antibodies used	Anti-GFP Antibody, Abcam, Cat# ab6658<br>Anti-Myc Antibody, Abcam, Cat# ab34773<br>Anti-DIG Antibody, Roche, Cat# 11333089001<br>Anti-mouse IgG Antibody, Jackson Immuno Research, Cat# 115-035-003<br>Anti-tubulin Antibody, Serotec/Bio-Rad, Cat# MCA2047
Validation	Validation was provided by suppliers. No additional validation was carried out because the antibodies were used only for in vitro assays with purified proteins. The specificity of interactions was confirmed using control experiments in which the relevant proteins were omitted.

April 2018
