# Supplementary material for: Microtubule end conversion mediated by motors and diffusing proteins with no intrinsic microtubule end-binding activity
Source: Nat Commun. 2019 Apr 11;10:1673. doi: 10.1038/s41467-019-09411-7 (PMC6459870; doi:10.1038/s41467-019-09411-7)
Supplement: Supplementary file 4 — Description of Additional Supplementary Files [file 41467_2019_9411_MOESM4_ESM.pdf]

Description of Additional Supplementary Files

File Name: Supplementary Movie 1
Description: MT gliding on beads coated with CENP-E motors. In this and all other experimental movies, the GMPCPP-stabilized MTs are labeled with Hilyte-647, so they appear in red; green indicates coverslip-immobilized 1-µm beads coated with GFP-tagged proteins. Scale bar, 3 µm. In this movie, beads are coated with the motor domains of CENP-E. MTs are initially attached laterally, but when ATP is added they glide on the beads and detach quickly. Because CENP-E is a plus-end–directed motor, the MT plus-end is the last contact point between the gliding MT and the bead. Movie plays 65 times faster than actual speed.

File Name: Supplementary Movie 2
Description: MT wall-to-end transition by CENP-E kinesin and Ndc80 complex. MTs glide visibly more slowly on beads coated with a mixture of CENP-E motors and Ndc80 Broccoli than on beads coated with CENP-E alone (compare to Supplementary Movie 1). After the MT plus-end reaches the bead, it is retained rather than dissociating, often persisting for longer than the total imaging time. Movie plays 65 times faster than actual speed.

File Name: Supplementary Movie 3
Description: Modeling of MT gliding over a molecular patch with CENP-E motors. In this and all other computational movies, MT is depicted in green, motors in blue, and Ndc80 molecules in red; for ease of viewing, only five molecules of each type are included. The movie frames playing at 30 fps show results for one out of every 104 -time steps of the corresponding simulation, yielding motions 3.3-fold slower than those in the model. In this movie, the patch-immobilized motors bind randomly and step toward the MT plus-end, propelling the MT minus-end forward. When the MT plus-end arrives at the molecular patch, it pauses briefly, awaiting the dissociation of the last motor.

File Name: Supplementary Movie 4
Description: Modeling of MT diffusion over a molecular patch with Ndc80 molecules. Patch-immobilized Ndc80 molecules randomly bind, diffuse on the MT wall, and then unbind, collectively driving the MT diffusion. MT ends are sometimes seen in the vicinity of the patch, but on average the MT is centered because all interactions are thermally driven, and the motions are not biased.

File Name: Supplementary Movie 5
Description: Modeling of MT wall-to-end transition by CENP-E and Ndc80. This calculation combines CENP-E motors and Ndc80 molecules, which interact with the MT in essentially the same manner as in Supplementary Movies 3 and 4. Their force-dependent ensemble behavior leads to durable MT end retention, even though the model assumes no special interactions between these molecules and MT tips.

File Name: Supplementary Movie 6
Description: Dynamic coupling between MT plus-ends and the bead-immobilized CENP-E and Ndc80 proteins. The movie starts after unlabeled soluble tubulin and GTP are added to the chamber, in which brightly labeled GMPCPP-stabilized MTs were transported and underwent end attachment to beads coated with CENP-E and Ndc80. Two labeled MT segments are seen slowly moving away from the bead and quickly retracting back, implying that tubulin is incorporated and lost from the bead-bound MT plus-ends. Movie plays 30 times faster than actual speed. Scale bar, 3 µm.

File Name: Supplementary Movie 7
Description: Dynamic coupling between MT plus-ends and bead-immobilized CENP-E and Ndc80 proteins in high $MgCl_2$. This movie is analogous to Supplementary Movie 6, but the experiment was performed in a buffer containing 7 mM instead of 2 mM $MgCl_2$. The rate at which the bright MT segment moves away from the bead is elevated, consistent with a higher rate of tubulin incorporation at the bead-coupled MT plusend. Movie plays 30 times faster than actual speed. Scale bar, 3 µm.

File Name: Supplementary Movie 8
Description: MT wall-to-end transition by CENP-E kinesin and the Ska1 complex. A GMPCPP-stabilized MT glides on a bead coated with a mixture of CENP-E motors and Ska1 proteins, undergoing wall-to-end transition. However, the MT plus-end detaches several minutes after establishing end attachment, indicating a problem with maintenance. Movie plays 65 times faster than actual speed. Scale bar, 3 µm.

File Name: Supplementary Movie 9
Description: MT wall-to-end transition by CENP-E kinesin and EB1 protein, reverse end-towall transition, and lateral MT diffusion. GMPCPP-stabilized MTs glide on a bead coated with a mixture of CENP-E motors and EB1 proteins, undergoing wall-to-end transition. After a few minutes, one of these MTs (labeled MT1) loses end attachment and undergoes lateral diffusion. CENP-E then returns the MT plus-end to the bead, and end coupling is re-established. Movie plays 65 times faster than actual speed. Scale bar, 3 µm.

File Name: Supplementary Movie 10

Description: MT wall-to-end transition by Kinesin-1 and Ndc80 proteins. GMPCPP-stabilized MTs bind to beads coated with a mixture of Kinesin-1 motors and Ndc80 proteins, glide rapidly, and detach almost immediately, indicating that they are unable to maintain end attachment. Movie plays 65 times faster than actual speed. Scale bar, 3 µm.
